# Ibuprofen-Induced Pancytopenia and Erythema Multiforme in an Elderly Female Patient

**DOI:** 10.7759/cureus.62785

**Published:** 2024-06-20

**Authors:** Stefan A Longobardi, Hamza Alkowati, Grace Kang, Cole Slade, Olu Oyesanmi

**Affiliations:** 1 Internal Medicine, Hospital Corporation of America (HCA) Florida Blake Hospital, Bradenton, USA; 2 Internal Medicine, Hospital Corporation of America (HCA) Healthcare Oak Hill Hospital, Brooksville, USA

**Keywords:** non-steroidal anti-inflammatory drugs, steven johnson's syndrome, toxic epidermal necrolysis, nikolsky's sign, ulcers, myelosuppresion, erythema multiforme, pancytopenia, ibuprofen

## Abstract

Erythema multiforme (EM) is a delayed, cell-mediated cutaneous disease with varying clinical manifestations. It is most commonly associated with infections but can also be associated with medications, vaccines, and autoimmune diseases. Non-steroidal anti-inflammatory Drugs (NSAIDs) are commonly used analgesics that have rare associations with EM and pancytopenia. These adverse reactions to NSAIDs can obscure definitive diagnosis due to their rarity. We present a case where an elderly female patient taking 600mg of ibuprofen up to four times a day for shoulder bursitis developed EM and pancytopenia. In this case, a 75-year-old female with a medical history of atrial fibrillation, essential hypertension, non-insulin-dependent type 2 diabetes mellitus, and ischemic stroke with residual right-sided visual impairment presented to our Emergency Department in 2023 with neck swelling, skin rash, and ulceration of the oral cavity. She reported a generalized, targetoid body rash that occurred 15 days after she started taking ibuprofen regularly for left shoulder bursitis.

No other medications were started before, after, or during this time period. CBC on admission was remarkable for a white blood cell count of 1.5x10^9^/L, hemoglobin of 6.5 g/dL, and platelet count <10x10^9^/L, consistent with pancytopenia. Ibuprofen was discontinued, and the patient was treated supportively with analgesia and packed red blood cell transfusions. Testing for HIV, antinuclear antibodies (ANA) panel, Hepatitis panel, and copper and zinc levels were negative. A biopsy of a targetoid lesion on the skin showed changes consistent with EM. Esophagogastroduodenoscopy revealed no actively bleeding lesions or ulcers in the stomach mucosa. The patient's blood counts eventually recovered with supportive treatment, and symptomatology improved. The patient was discharged six days after admission. Healthcare professionals should be aware of rare hematologic and immunologic side effects of NSAIDs, which may often be overlooked and misdiagnosed. More studies are needed to build on our wealth of knowledge regarding the etiology and management of EM, Steven Johnson syndrome (SJS), and toxic epidermal necrolysis (TEN).

## Introduction

Erythema multiforme (EM) is a delayed, cell-mediated cutaneous disease with a wide and varying clinical involvement. EM is most commonly associated with infections such as Herpes simplex virus (HSV) 1 and 2, Mycoplasma pneumoniae, and Histoplasma capsulatum. On the other hand, vaccinations, autoimmune diseases, and medications (Non-steroidal anti-inflammatory drugs (NSAIDs), Allopurinol, Sulfonamides, Aminopenicillins, Nitrofurantoin, and Tetracycline [1]) are more rare causative factors. Ibuprofen can cause acute generalized exanthematous pustulosis (AGEP), drug rash with eosinophilia and systemic symptoms (DRESS), and Stevens-Johnson syndrome (SJS), however, its association is <0.1% with EM [2]. Primary drug-induced EM is typically confined to the oral mucosa, but recurrent episodes may produce more severe forms of variable skin and mucosal membrane involvement [3]. Ibuprofen may also cause a reduction of bone marrow myelopoiesis via immunological mechanisms, and there are few cases of agranulocytosis reported [4]. Due to the high availability of ibuprofen worldwide, it is important to recognize and distinguish cutaneous hypersensitivity reactions and blood dyscrasias linked to ibuprofen (NSAIDs).

## Case presentation

**Figure 1:**
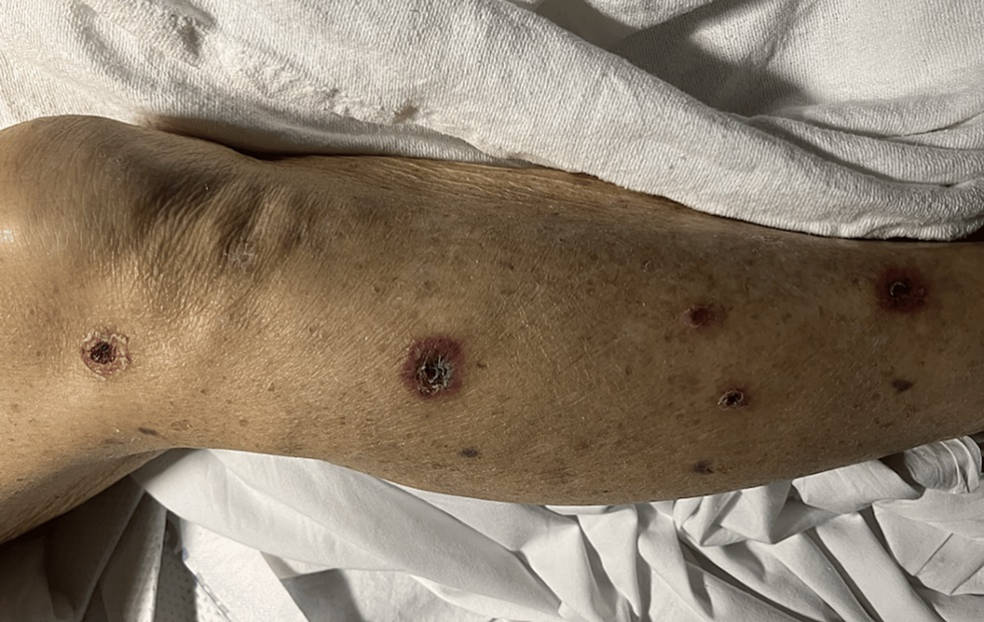
Targetoid circular plaques with central heme crusting and necrosis

**Figure 2:**
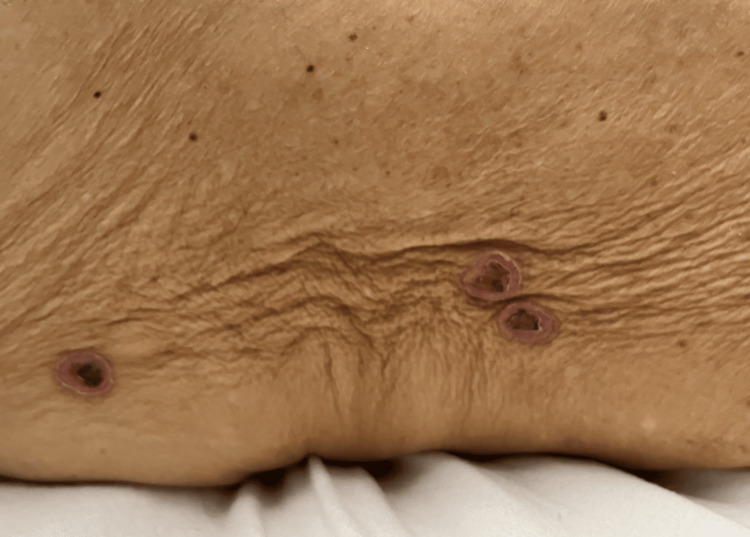
Targetoid circular lesions with central crusting and necrosis

**Figure 3:**
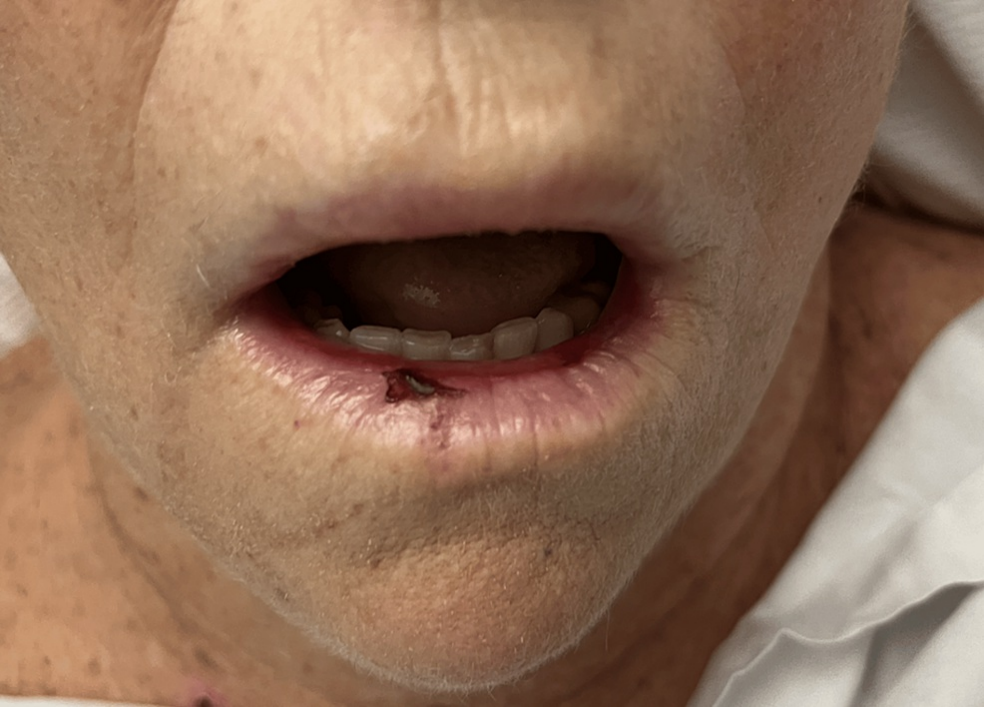
Oral mucosal ulcer

A 75-year-old female with a medical history of atrial fibrillation, essential hypertension, non-insulin-dependent type 2 diabetes mellitus, and ischemic stroke with residual right-sided visual impairment presented to our Emergency Department in 2023 with neck swelling, skin rash, and ulceration of the oral cavity. She reported a generalized body rash that occurred 15 days after she started taking 600mg of ibuprofen up to four times daily regularly for left shoulder bursitis. Over the next three days, her symptoms worsened. She began to notice an increased amount of bleeding from the oral ulcers as well as an aversion to food and weight loss secondary to odynophagia. She started having black tarry stools, prompting her to seek medical attention. Upon ENT examination, the patient had scattered oral mucosal ulcers and generalized fragile fluid-filled vesicles (unable to be captured in the photograph). Examination of her skin was significant for diffusely scattered targetoid circular lesions with central heme crusting and necrosis present on the trunk, arms, and legs (Figures 1-3). 

She stopped her home rivaroxaban after she noticed bleeding from her mouth, and she was non-compliant with the rest of her long-term home medications due to odynophagia. She did not change any other medications within these 15 days. Her only allergy was to erythromycin, from which she developed anaphylaxis. Other medications that she used regularly and chronically (>1 year) included hydrochlorothiazide, lisinopril, glipizide, metformin, and propranolol. The patient had a noncontributory family history of coronary artery disease and diabetes mellitus. She did not smoke or use alcohol. Past surgical history included hysterectomy with bilateral salpingo-oophorectomy and parathyroidectomy.

Upon presentation, her vital signs were significant for sinus tachycardia. She was afebrile, with a normal respiratory rate, blood pressure, and oxygen saturation on room air. Complete blood count (CBC) was significant for a white blood cell count of 1.5x10^3^/µl (Reference Range: 4.5-10.5x10^3^/µl), hemoglobin of 6.5 g/dL (Reference Range: 11.2-15.7g/dL), as well as platelet count <10x10^3^/µl (Reference Range: 150-450x10^3^/µl) (Table 1). The white blood cell count had a lower proportion of neutrophils (18.2%, normal: 34.0-71.1%) and a higher percentage of lymphocytes (72.7%, normal: 19.3-51.7%) and eosinophils (6%, Normal: 1-3%). The comprehensive metabolic panel was within normal limits. Initial computed tomography of the abdomen and pelvis with contrast and chest x-ray were negative for any acute pathology. She was admitted to the hospital for further management.

**Table 1:** Relevant complete blood count values during admission. *Indicates the days when the patient was transfused 1 unit of packed red blood cells

Day of Hospitalization	White Blood Cell Count (x10^3^/µl) (Reference Range: 4.5-10.5x10^3^/µl.)	Hemoglobin (g/dL) (Reference Range: 11.2-15.7 g/dL)	Platelet Count (x10^3^/µl) (Reference Range: 150-450 x10^3^/µl)
Day 1	1.5	6.5*	<10
Day 2	0.7	6.8*	11
Day 3	1.8	6.9*	67
Day 4	2.5	7.5	62
Day 5	2.8	6.9*	87
Day 6	3.6	8.3	136
Day 7	4.3	9.3	210

**Figure 4:**
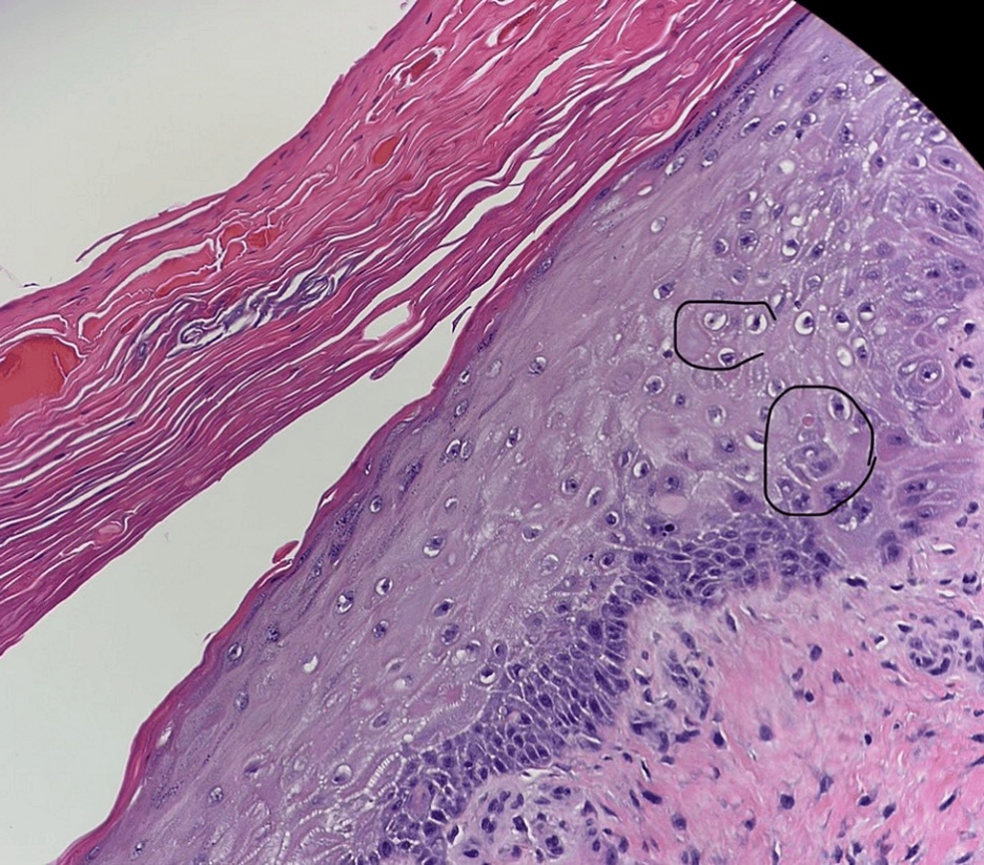
Skin biopsy showing mild epidermal spongiosis and rare dyskeratotic keratinocytes

Throughout her 6-day hospital course, we treated the patient supportively with intravenous fluids, pain medications, pantoprazole for GI bleed, and a total of 4 transfusions of packed red blood cells. We held her home with rivaroxaban and ibuprofen. No steroids were administered other than one dose of dexamethasone in the ED. As part of the workup for pancytopenia, we ordered a hepatitis panel, HIV test, vitamin B12 level, folate level, and antinuclear antibodies (ANA) pattern, which were all negative. On day two of her hospitalization, we performed two punch biopsies of the skin lesions measuring 0.3cm in diameter x 0.3cm in depth. The pathology showed features suggestive of resolving EM and/or Steven Johnson's Syndrome (Figure 4). We also consulted Gastroenterology for endoscopic evaluation in the setting of upper gastrointestinal bleeding. Subsequent esophagoduodenoscopy failed to reveal any ulcerated, bleeding lesions. The esophagoduodenoscopy revealed stomach mucosal changes consistent with gastritis. Biopsy of the stomach and duodenum was negative for Helicobacter pylori and malignancy.

The supportive medical treatment resulted in a gradual improvement in her CBC and her cessation of the previously reported black and tarry stools. She had less bleeding and marked improvement in her oral ulcer and skin lesion symptomatology. Upon discharge (day 6 post-admission), her white blood cell count was 4.3x10^3^/µl, hemoglobin was 9.3 g/dL, and platelet count was 210x10^3^/µl (Table 1). We discharged her with return precautions and strict instructions to avoid the use of ibuprofen and any other NSAIDs.

## Discussion

Erythema multiforme is a manifestation of the skin and oral mucosa that usually presents as symmetric target-like lesions caused by an immune-mediated response. Most commonly, EM is secondary to an infection such as herpes simplex virus, mycoplasma pneumoniae, and certain medications. Erythema multiforme may present a spectrum of severity. It is subdivided into a less severe form named erythema multiforme minor and a more severe form named erythema multiforme major. The two forms are similar in the morphology of the cutaneous lesions; however, they differ in the amount of mucous membrane involvement. Erythema multiforme minor has minimal mucosal involvement as compared to erythema multiforme major.

In many instances, EM major can be misdiagnosed as Steven-Johnson syndrome. Some sources argue that the two are not separate disease entities; rather, they are a spectrum of a singular disease process [5,6]. Notably, Steven-Johnson syndrome presents as widespread painful purpuric macules and plaques on the trunk or atypical target lesions. These said lesions are often dusky. Erythema multiforme major generally presents on the extremities in a symmetric pattern without blistering [5]. Steven-Johnson syndrome commonly evolves into flaccid bullae, which are Nikolsky's positive signs, causing large and painful erosions [6].

Additionally, the denudation in erythema multiforme major is generally limited to 1-2% of the body surface area, while Steven Johnson syndrome can involve up to 10% of the body surface area. Over 30% of body surface area involvement is classified as toxic epidermal necrolysis (TEN) [6]. Between 10-30% of body surface area involvement is considered Steven Johnson/toxic epidermal necrolysis overlap.

The clinical picture varies in patients with EM involvement, including oral mucosa, skin, or a mixture of both. Nonspecific symptoms such as headache, fever, sore throat, and cough can precede any clinical picture of skin lesions. The estimate of mucous membrane involvement is typically around 25 to 60%; the oropharynx, lips, and conjunctiva are the most frequently and severely affected. The most well-recognized presentation is the abrupt onset of symmetrically distributed erythematous macules and papules that grow into bullous, gyrate, vesicular, annular, or iris-shaped lesions that may become hemorrhagic [7]. Our patient reported days of ulcerated lesions of the oral cavity with positive Nikolsky's sign followed by symmetric targetoid skin lesions on the limbs. In this case, there was considerable overlap in presentation between EM and Steven-Johnson syndrome, making the initial diagnosis difficult to establish. As aforementioned, Steven-Johnson syndrome tends to affect more surface area, is associated with more denudation of the epithelium, has more truncal localization, and has a paucity of the typical "target lesion" [8]. This helped us narrow our differential diagnosis to EM, as our patient had more cutaneous limb involvement with denudation limited to the oral cavity. The development of severe pancytopenia also confounded the differential diagnosis by causing profuse bleeding from cutaneous sites.

Erythema multiforme is typically diagnosed clinically using a thorough history and physical examination. The search for the underlying etiology can be challenging. Common pharmacological causes of EM include NSAIDS, antibiotics, and anticonvulsants [9]. Further laboratory investigations may be necessary, i.e., HSV testing, autoimmune workup, and other serological tests. A skin biopsy should be performed when there is uncertainty in establishing a definitive diagnosis. Biopsy typically shows epithelial intercellular edema (spongiosis) with keratinocyte necrosis, which is responsible for an intra- or sub-epidermal blister and vacuolar interface dermatitis. An important differentiating factor from Steven Johnson syndrome is that EM has more cellular infiltrate and dermal inflammation. Our patient's biopsy was consistent with EM and showed necrotic keratinocytes.

Mild anemia, thrombocytopenia, and leukocytosis may also be seen in patients with EM [10]. On the contrary, our patient had severe pancytopenia on her complete blood count. This was likely secondary to a rare idiosyncratic drug reaction of NSAIDs that resulted in pancytopenia. Our case was unique in that it showcased two rare side effects of NSAIDs (pancytopenia and EM) despite correct medication usage. Ibuprofen is considered one of the safest NSAIDs in terms of side effect profiles [11]. Only a few cases of neutropenia, thrombocytopenia, and hemolytic anemia have been reported. The mechanisms by which NSAIDs affect the hematopoietic system have been described as a possible antigen-antibody reaction regarding the drug/drug metabolite and the membrane of the blood cell, of which destruction can occur in the periphery as well as the bone marrow [8]. The side effects may also be related to the excessive use of the medication in a susceptible patient, which is unlikely to be the case in our patient as she was taking the medication as prescribed. The vast majority of the hematological side effects of NSAIDs are secondary to immunological mechanisms [8]. The probability of an adverse drug reaction in our patient was 7 out of 10 according to the Naranjo scale, which is a questionnaire that assesses the likelihood of a drug causing an adverse reaction [12]. This represents that the adverse reaction is probable. Although we do not have a bone marrow biopsy, our patient did not change any medications prior to, during, or after the usage of ibuprofen. Her skin lesions and blood counts improved with supportive treatment and discontinuation of ibuprofen. Otherwise, the clinical picture could not be reasonably explained with her known clinical state.

The reported epidemiologic data related to EM is scarce. This is likely because of the acute nature of the condition, the absence of a universally accepted classification system, and the lack of a reporting registry. The reported prevalence rate of EM is less than 1% [13]. EM typically occurs in young adults 20 to 40 years of age and is more common in women [13]. Patients who are immunocompromised, immunosuppressed, or have underlying autoimmune disorders are more predisposed to developing EM [13]. Although EM due to drugs is rare, the drugs that are most commonly associated are NSAIDs and antibiotics. Oral lesions can occur in 70% of cases [14]. Our patient presented with oral lesions as well as cutaneous lesions. She presented outside of the typical age range. The histopathological and clinical manifestations of EM can be characteristic but not specific. For example, in an international case-control study conducted in Europe (SCAR), 92 out of 552 patients could not be classified as EM or SJS [15]. In this same study, the etiologic fraction of drugs traced to EM was 5%, as compared to 45%+ in Steven-Johnson syndrome, which again illustrates the rarity of drug-induced EM. Although EM has a variable disease course, it is mostly a self-limited disease lasting from a couple of days to one month, as was the case with our patient.

The management of EM is difficult because of the varying etiologies and differing clinical presentation of the disease. The primary literature is sparse, with few to no randomized controlled trials. Any possible offending agents are removed and treated accordingly. For example, EM secondary to herpes would be treated with acyclovir; in the event the patient is infected with mycoplasma, tetracycline may be used. Acute mild-moderate EM, due to its largely self-limiting nature, can be treated with topical corticosteroids and antihistamines [15]. Severe EM should be treated in the hospital setting, with IV fluids and steroids such as prednisone 40-60 mg/day tapered over two weeks [15]. Alternatively, treatment can also be achieved with weight-based dosing of prednisone (0.5-1mg/kg/day tapered over two weeks) [16]. However, there is still debate regarding the most beneficial steroid, its dose, duration, and its route of administration. Methylprednisolone 1mg/kg/day IV for three days has shown efficacy, as has dexamethasone pulse therapy (1.5mg/kg IV on three consecutive days) [16,17]. On the contrary, one source suggests tapering oral prednisolone over one week, while another suggests high-dose steroids followed by a one-month taper [18,19]. Regardless of the specific suggestion, it is clear that early administration of systemic steroids in severe EM provides significant benefits.

Erythema multiforme major must be monitored for the evolution of the cutaneous lesions that might suggest SJS, such as increased denudation. These patients may need to be managed in a specialized burn unit. Ophthalmologic consultation may be beneficial if there is ocular involvement. HSV is the most common etiologic agent for recurrent EM, and the first-line treatments are antivirals. In the few existing case studies, there seems to be some efficacy regarding using immunomodulators such as rituximab and anti-TNFα inhibitors [20,21]. Thalidomide, azithromycin, and dapsone have also shown potential [22,15]. In our patient, we removed the offending agent and initiated supportive care with mouthwash and gastric protection in the form of pantoprazole. We observed our patient for worsening of her skin lesions. We did not use steroids despite the patient having ulcers with mucosal involvement and blood loss anemia. The patient showed rapid clinical improvement by discontinuing the potential culprit agent (ibuprofen). Esophagogastroduodenoscopy revealed gastritis, and no intervention was needed for active bleeding.

## Conclusions

Healthcare professionals should be aware of rare hematologic and immunologic side effects of NSAIDs, which may often be overlooked and misdiagnosed. More studies are needed to build on our wealth of knowledge regarding EM, SJS, and TEN. In severe cases, steroids remain the mainstay of treatment; however, other immunomodulating agents show promise. This presentation of EM favors the use of skin biopsy to help differentiate between more concerning dermatologic pathologies such as SJS or TEN because of the overlap in clinical presentation. This biopsy approach helped to avoid the unnecessary use of steroids and instead manage the patient with supportive care.
